# Ear and hearing care programs for First Nations children: a scoping review

**DOI:** 10.1186/s12913-023-09338-2

**Published:** 2023-04-19

**Authors:** Kai Nash, Rona Macniven, Liesa Clague, Harvey Coates, Mark Fitzpatrick, Hasantha Gunasekera, Kylie Gwynne, Luke Halvorsen, Samantha Harkus, Leanne Holt, Noeleen Lumby, Katie Neal, Neil Orr, Elizabeth Pellicano, Boe Rambaldini, Catherine McMahon

**Affiliations:** 1 The Djurali Centre for Aboriginal and Torres Strait Islander Health Research and Education, Sydney, Australia; 2 School of Population Health, University of New South Wales, Sydney, Australia; 3 Thurru Indigenous Unit, College of Medicine, Health and Wellbeing, University of Newcastle, Newcastle, Australia; 4 The University of Western Australia, Perth, Australia; 5 Telethon Speech and Hearing, Perth, Australia; 6 The University of Sydney, Sydney, Australia; 7 National Acoustic Laboratories, Sydney, Australia; 8 Department of Indigenous Studies, Macquarie University, Sydney, Australia; 9 The Shepherd Centre, Newtown, Australia; 10 University College London, London, UK

**Keywords:** Indigenous, Aboriginal, Māori, Inuit, Metis, Middle ear disease, Hearing, Healthcare

## Abstract

**Background:**

Ear and hearing care programs are critical to early detection and management of otitis media (or middle ear disease). Otitis media and associated hearing loss disproportionately impacts First Nations children. This affects speech and language development, social and cognitive development and, in turn, education and life outcomes. This scoping review aimed to better understand how ear and hearing care programs for First Nations children in high-income colonial-settler countries aimed to reduce the burden of otitis media and increase equitable access to care. Specifically, the review aimed to chart program strategies, map the focus of each program against 4 parts of a care pathway (prevention, detection, diagnosis/management, rehabilitation), and to identify the factors that indicated the longer-term sustainability and success of programs.

**Method:**

A database search was conducted in March 2021 using Medline, Embase, Global Health, APA PsycInfo, CINAHL, Web of Science Core Collection, Scopus, and Academic Search Premier. Programs were eligible or inclusion if they had either been developed or run at any time between January 2010 to March 2021. Search terms encompassed terms such as First Nations children, ear and hearing care, and health programs, initiatives, campaigns, and services.

**Results:**

Twenty-seven articles met the criteria to be included in the review and described a total of twenty-one ear and hearing care programs. Programs employed strategies to: (i) connect patients to specialist services, (ii) improve cultural safety of services, and (iii) increase access to ear and hearing care services. However, program evaluation measures were limited to outputs or the evaluation of service-level outcome, rather than patient-based outcomes. Factors which contributed to program sustainability included funding and community involvement although these were limited in many cases.

**Conclusion:**

The result of this study highlighted that programs primarily operate at two points along the care pathway—detection and diagnosis/management, presumably where the greatest need lies. Targeted strategies were used to address these, some which were limited in their approach. The success of many programs are evaluated as outputs, and many programs rely on funding sources which can potentially limit longer-term sustainability. Finally, the involvement of First Nations people and communities typically only occurred during implementation rather than across the development of the program. Future programs should be embedded within a connected system of care and tied to existing policies and funding streams to ensure long term viability. Programs should be governed and evaluated by First Nations communities to further ensure programs are sustainable and are designed to meet community needs.

**Supplementary Information:**

The online version contains supplementary material available at 10.1186/s12913-023-09338-2.

## Background

First Nations people, a collective term used here to describe the original inhabitants of Australia, New Zealand, Canada, and the United States,^1^ have their own unique tribes, languages, cultures, traditions, and histories. First Nations people have endured systemic loss of culture and language, dispossession of traditional lands, and marginalisation [1], yet, have also have demonstrated extraordinary resilience to impacts of colonisation [2]. However, one of the remnants of colonialisation is social and historical determinants of health which continue to drive disparities in social and health outcomes [3, 4].

First Nations people in Australia, New Zealand, Canada, and the United States present with below average life expectancy and poor health [5–17], despite residing in highly-developed countries with government commitment to health equity [18–22]**.** Among these health disparities, otitis media (middle ear disease resulting from upper respiratory tract infections) presents in First Nations children with some of the highest prevalence rates in the world [23–28]. First Nations children endure longer [26] and more severe bouts of infection [26, 27], and are at increased risk of associated complications [26, 27]. Acquired hearing loss is associated with such infections, particularly in the case of chronic suppurative otitis media (CSOM) whereby chronic middle ear inflammation and perforation of the tympanic membrane can result in permanent hearing loss [29].

Even mild levels of hearing loss can have a significant impact on communication of young school-aged children [30]. In turn, these difficulties can hinder learning and academic attainment [30, 31]. Hearing loss and social disadvantage are interconnected [32, 33] and the compound effect is likely to be significant [34, 35]. Hearing is a key factor in speech language development [36], cognition and social communication [36], academic pursuits [30, 31], wellbeing [37]**,** behavioural skills [38, 39], social ease [40]**,** employment, and socio-economic status later in life [41]**.** Hearing is also an integral part of learning, culture, and storytelling for First Nations people [42–45].

Otitis media is classified as a ‘wicked problem’ in First Nations people. Otitis media is an issue of complexity and is particularly challenging to solve among First Nations populations owing to social and historical determinants of health such as overcrowded living conditions and low socio-economic status [46, 47]. These impacts are further compounded by the lack of accessible ear and hearing care services available to First Nations children [48], in part due to systemic and structural racism embedded in healthcare in many of these countries [49]. Culturally safe health services for First Nations people have emerged to address challenges of discrimination in healthcare settings [50]. Cultural safety is an ongoing process involving reflection of beliefs, biases, and stereotypes, acknowledging and addressing these perceptions with the aim of providing culturally safe care as defined by the patient and wider community [51]**.**

Programs providing early detection and timely management of otitis media and associated hearing loss are fundamental to preventing downstream impacts on health, social, and educational outcomes. Yet, to date, little is known about the ways in which ear and hearing care programs that exist for First Nations children have been designed to mitigate barriers to accessing care. Therefore, this scoping review aimed to identify ear and hearing care programs for First Nations children in high-income colonial-settler countries to; (i) chart program strategies, and (ii) map the programs according to care pathway focus. This review also aimed to identify reported factors of program sustainability and measures of program success.

## Methods

In late 2020, the Aboriginal Children’s Hearing Health Project team identified the need to better understand approaches, areas of focus, and sustainability factors for healthcare programs that focus on ear and hearing issues among First Nations children in high-income colonial-settler countries. Ten non-Aboriginal members (RM, HC, MF, HG, KG, SH, KNeal, NO, EP, and CM) of the Aboriginal Children’s Hearing Health Project and six Aboriginal and Torres Strait Islander members (KNash, LC, LHolt, LHalvorsen, NL, BR) made significant contributions to the review with expertise in scoping reviews and Aboriginal and Torres Strait Islander healthcare, culture, and lived experience.

The current scoping review was conducted in accordance with Joanna Briggs Institute methodology [52].

### Eligibility criteria

The database search was conducted in March 2021 and programs were eligible for inclusion if they had either been developed or run at any time between January 2010 to March 2021. Programs which were not developed or operational at least partially within this time was excluded on the basis that these programs do not capture recent approaches to ear and hearing care for First Nations children. Studies both qualitative and quantitative were included if they described healthcare programs that focused on ear and hearing problems for First Nations children aged up to 12 years of age in high-income colonial-settler countries, namely Australia, New Zealand, Canada, and the United States. Studies describing ear and hearing care programs which did not service First Nations children were excluded. Health awareness programs and campaigns regarding ear and hearing care were included if the targeted population was parents, carers, early childhood educators, teachers or health professionals who had First Nations children under their care.

### Information sources

A Macquarie University librarian was consulted to compile the search strategy with MeSH terms and key word phrases including search terms such as ear and hearing care, First Nations, and health programs. Eight electronic databases were systematically searched: Medline, Embase, Global Health, APA PsycInfo, CINAHL, Web of Science Core Collection, Scopus, and Academic Search Premier. Grey literature including Google, Google Scholar, and Indigenous HealthInfoNet were also searched. References from included studies was scanned to identify additional studies.

### Search

The full search strategy used to search the Medline database is provided in Table 1. The search strategy used for Medline was modified as required and used as the basis for the other database searches.

**Table 1:** Medline search strategy

No	Search terms
1	Indigenous Peoples/
2	Oceanic ancestry group/ or Indians, North America/
3	Inuits/
4	(Indigenous or aborigin* or Torres Strait Island* or maori* or inuit* or alaskin native* or Eskimo* or American Native* or American Indian* or Native American* or Metis* or First nation* or First people*).ab,ti
5	or/1–4
6	"Delivery of Health Care"/
7	Health Services/
8	Primary Health Care/
9	((healthcare or health care or medical*) adj3 (deliver* or service* or community or primary)).ab,ti
10	(healthcare or health care or primary health or model or service delivery or medical service* or health service*or community care* or community health*).ab,ti
11	or/6–10
12	((hearing or hear or audi* or aural* or ear) adj3 (impair* or loss* or disab* or dysfunct* or problem* or screen* or treatment* or rehab* or interven* or program* or outcome* or service* or health* or nose)).ab,ti
13	Hearing Loss/
14	Hearing Disorders/
15	Otitis Media/
16	or/12-First Indigenous Peoples/
17	5 and 11 and 16
18	Limit 17 to (English and yr = ”2010–2021″)

### Selection of sources of evidence

Two independent reviewers (KN and RM) screened by abstract and title and then screened by full text. The reviewers critically appraised eligible programs, and one independent reviewer (KN) extracted data from included studies. Any disagreement was settled with discussion and inclusion of a third reviewer (CM).

### Data charting process

The content of each article was extracted with reference to characteristics of ear and hearing care programs described including approaches, areas of focus across the care pathway, and sustainability factors. Indicators which studies reported on regarding program success were also captured. Data extraction was conducted by one of the independent reviewers (KN) and verified by a second independent reviewer (RM). Any disagreements were resolved through discussion with a third reviewer (CM).

### Data items

The data extraction spreadsheet contained the following items: First author, program/activity name, program/activity details, care pathway focus, years active, state and country, setting, participants, approach, access challenges, solutions to access challenges, First Nations involvement in design, implementation or evaluation, sustainability factors, indicators used to identify program success.

### Synthesis of results

Data regarding programs were reported using descriptive and narrative synthesis with reference to program care pathway focus, years active, state and country, setting, participants, program/activity details, program approach, access challenges, solutions to access challenges, First Nations involvement in design, implementation or evaluation, sustainability factors and indicators used to identify program success.

Programs typically were focussed on addressing specific barriers along the care pathway. Therefore, these were mapped onto one or more of four parts of a typical care pathway (prevention, detection, diagnosis / treatment, and rehabilitation). For the purposes of understanding care pathways within the ear and hearing care context, prevention was conceptualised as including education/awareness campaigns on prevention of speech and language delay, middle ear disease, and hearing loss. Detection included awareness of ear disease and hearing loss symptoms, surveillance, and screening. Diagnosis was the determination of condition or severity of the condition. Management included ENT surgery and medical treatment such as antibiotics. As diagnosis and management often occur within the same service or are carried out by the same provider, these two components of the care pathway have been conceptualised as occurring at the same care pathway stage. Finally, rehabilitation was conceptualised as involving learning support in schools, speech therapy and counselling as well as hearing aids and cochlear implants.

## Results

### Selection of sources of evidence

**Fig. 1:**
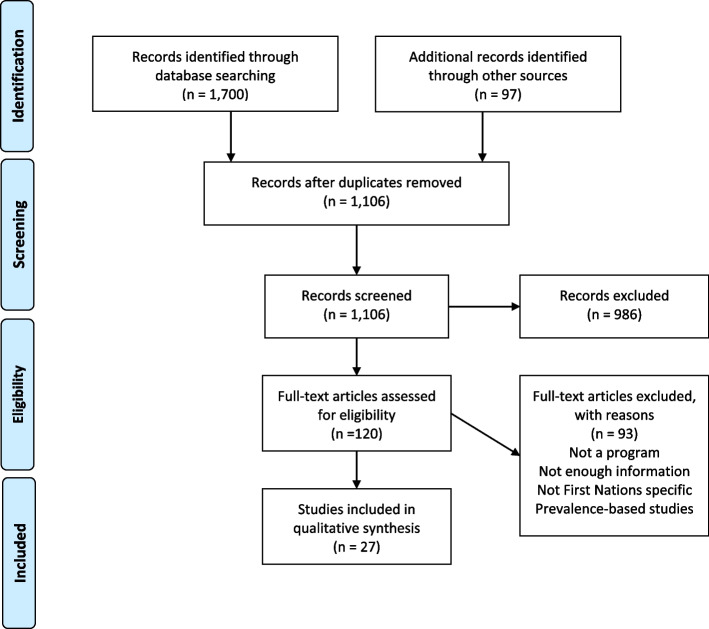
PRISMA flow chart

The formal search identified 1,700 studies. Grey literature searches identified a further 97 studies. There were 1,106 studies remaining after duplication removal and these records were all screened by abstract/title. There were 120 studies assessed by full text according to eligibility criteria and 93 of these studies were removed with stated reasons. Twenty-seven studies were included in the synthesis. Study selection follows the PRISMA reporting guidelines and is illustrated in Fig. 1 [53].

### Characteristics of sources of evidence

Identified articles (*N* = 27) were published between December 2010 and February 2021. Eight articles were classified as descriptive reports [18, 54–60], while the remaining 19 have been classified as studies [20, 21, 42, 61–76]. Of the 19 studies, two were cross sectional [74, 75], five were observational/descriptive [20, 21, 42, 66, 69]**,** five were retrospective [62, 70–73], three were qualitative [61, 65, 76], three were cost-effectiveness analyses [63, 64, 67] and one was a randomized controlled trial [68]. It should be noted that the randomized controlled trial was eligible for inclusion due to the intervention/program approach taken, while prevalence studies were excluded from this scoping review due to only providing prevalence data.

### Program location

Twenty-one programs were reported across 27 studies. Programs were active between 1985 – 2021. Most programs were conducted in Australia (*N* = 18), one program was active in Canada (*N* = 1), and two programs were active in Alaska (*N* = 2). No programs were identified as being active in New Zealand. Supplementary Table 1 captures an overview of the ear and hearing care program activities.

### Care pathway focus

#### Mapping programs onto the care pathway

**Fig. 2:**
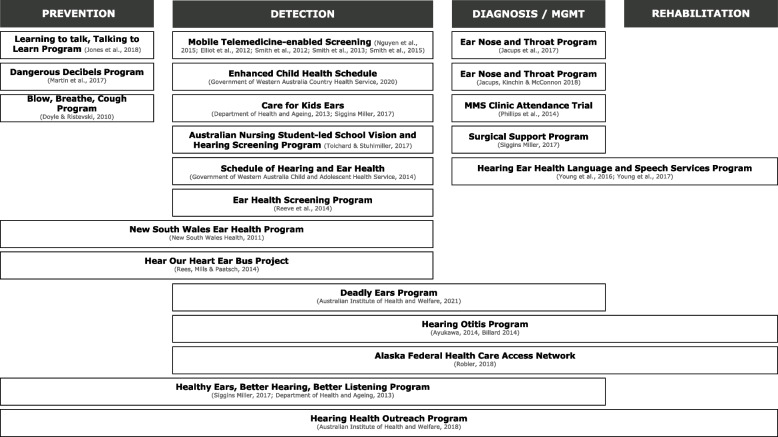
Mapping programs onto the care pathways

Understanding the care pathway is essential to ensuring continuity of care through identifying treatment processes and timeframes [77]. Mapping programs to the care pathway (see Fig. 2) may help identify which areas of the care pathway are being concentrated on and what areas may require further attention. It also highlights where the major barriers might be in accessing the pathway. Three programs focused exclusively on prevention, while others (*N* = 4) included, although not exclusively, prevention strategies. Thirteen programs focussed on detection, most of which related to screening of otitis media and hearing loss (*N* = 11). Ten programs were identified as focussing on management, which involved medical management and ENT specialist services. Four programs contained a rehabilitation component. Of the 21 programs, eight focused on more than one part of the care pathway.

### Program strategies

#### Key barriers which programs aimed to address

The two most commonly identified barriers to accessing existing (mainstream) services were geographical [18, 20, 21, 42, 56, 58, 60, 62–64, 69–73] and low levels of awareness of how to prevent the impacts of ear disease and hearing loss in young children as well as strategies to mitigate impacts [18, 20, 21, 56, 58, 63, 65, 66, 68, 70–73, 75, 76]. These barriers were either specifically reported in publications relating to the program or inferred through specific program strategies (e.g. it was assumed that implementation of telehealth in regional and remote areas indicated a geographical barrier to services). Additional barriers identified included extensive ENT waitlist times [18, 58, 64, 65, 70, 75, 76], lack of service coordination [18, 58], system fragmentation [67], workforce shortages [58], implementation difficulties [74], and cost [18, 20, 21, 64]. Further, coordinating access to tertiary care was identified as challenging due to systemic barriers [18]. Studies identified lack of awareness of availability of existing programs [61], ear and hearing health issues [18, 55, 66, 69], low clinic attendance [67, 68], and poor adherence to treatment plans [68].

#### Extend geographical reach of services (connecting people to care)

##### Telehealth

Eight programs employed telehealth to extend geographical reach of services and connect patients to timely care. The mobile screening and surveillance service in Queensland (QLD) Australia [62, 67, 71–73], the Hearing Health Outreach Program in the Northern Territory (NT) Australia [58] and an ear health screening program in New South Wales (NSW) Australia [70] employed asynchronous telehealth. The Deadly Ears program in QLD Australia [60] utilised asynchronous telehealth to support delivery of ENT services, nursing and allied health services. The Alaska Federal Health Care Access Network (AFHCAN) program utilised synchronous and asynchronous telehealth as facilitated through community health aids liaising with specialists who manage care remotely. Only, if necessary, a patient may be expedited to receive in-person treatment thus bypassing delays [42].

An ENT program in Australia (no name) utilised telehealth for post-operative review to address geographical challenges and deliver a cost-effective model of care [64]. This service model for ENT surgery required the patient to travel to a regional centre for surgery. A different study describing this system of care also describes two alternative models which would utilise telehealth to improve accessibility [63]. The Hearing Health Outreach Program in the NT also utilised telehealth to reduce patient travel times for ENT services [58].

##### Outreach

Five programs were identified as employing an outreach approach to connect services to communities in regional or remote locations. The Hearing Health Outreach Program utilised an outreach approach to offer rehabilitative services [58]. Healthy Ears—Better Hearing, Better Listening program prioritized services to locations of highest need through utilizing an outreach approach [18, 56]. The Australian program Enhanced Child Health Schedule (ECHS) offered additional home visiting contacts for families considered to have high priority needs [59]. Hear our Heart Ear Bus Project (HoHEBP) [69] and Mobile screening and surveillance service in Australia [62, 67, 71–73] utilised mobile screening clinics to extend geographical reach. The Surgical Support program offered financial support to cover travel and accommodation expenses for both health professionals and patients and their carers [18].

##### Other

The Hearing and Otitis Program (HOP) in Nunavik Canada was established in a specific location in the north in response to geographical barriers. This program also offered to send individual’s hearing aids to specialists via mail [20, 21]. The Australian Nursing Student-led School Vision and Hearing Screening Program employed a community-level approach to reduce the need for travel to access primary care services [74]. The AFHCAN program is a state-wide telehealth/telepractice network which integrated into clinical practice at 248 sites across the state thereby increasing specialty care access [42]. The Care for Kids Ears program was available online and Australia-wide for those who were able to access it [18, 55].

#### Ensuring cultural safety

##### Indigenous Health Worker (IHW) Involvement

Eleven programs were identified as employing IHWs to ensure cultural safety. The HOP in Nunavik Canada delivered hearing aid services such as fitting, follow-up and minor repairs facilitated through the program’s culturally identified role of the ‘aaniasiurtiapiit’ – a role similar to that of an IHW. If necessary, the aaniasiurtiapiit would send the hearing aid via mail to another specialist for further assistance. Counselling was provided by the siutilirijiit, a cultural counsellor who ensured safety of linguistic and cultural needs regarding proposed solutions to hearing loss [20, 21].

Aboriginal Health Workers (AHWs) provided clinical services, support, follow-up services or use of resource kits in seven programs including the Hearing Health Outreach Program [58], Healthy Ears—Better Hearing, Better Listening [18, 56] Care for Kids Ears [18, 55] mobile screening and surveillance service in QLD Australia [62, 67, 71–73], ECHS program [59], HoHEBP [69] and in the current service model for ENT surgery in the NT [63].

The Hearing Health Outreach Program in the NT delivered diagnostic services through outreach teams, which consisted of an audiologist and at least one other additional staff member (either a registered nurse, nurse audiometrist, AHW or community health worker) [58]. This outreach program included training of Aboriginal community hearing workers in hearing health education, promotion and prevention [58].

##### Skilled workforce trained in cultural safety

Three programs employed a workforce who had been trained in culturally safe work practices. The AFHCAN program aimed to ensure cultural safety through providing an audiologist who understands cultural subtleties of non-verbal communication such as facial movements and eye contact. This program also aimed to provide hearing technology choices that considered the patient’s cultural needs, which varied substantially due to diverse choices of lifestyle and environments including lakes, rivers and tundra [42]. The HOP involved capacity strengthening including training of local community members (95% of the residents of Nunavik are of Inuit ancestry) to take on roles in the hearing program [20, 21]. The AFHCAN program utilised culturally competent audiologists [42].The Hearing EAr health Language and Speech services (HEALS) project in NSW Australia, utilised existing Aboriginal Community Controlled Health Organization partnerships to ensure cultural safety of services [75, 76]. The ECHS program was developed in consultation with Aboriginal Health staff, refugee health staff, internal and external health experts [59]. Supplementary Table 2 captures ear and hearing care core program elements, including specific program strategies (e.g. outreach), solutions implemented to address challenges (e.g. telehealth to overcome geographical remoteness), First Nations involvement (in program design, program implementation and/or program evaluation), and identified program sustainability factors (e.g. funding).

##### First Nations people involvement in program design, implementation, and evaluation 

Self-determination is a key aspect of health services for First Nations communities, whereby community collaboration and design of heath programs ensures services are delivered in a manner that meets local needs and facilitates better health outcomes [50]. Of the 27 articles, seven did not report any First Nations people involvement. Most of the remaining studies reported First Nations people involvement in program implementation [18, 20, 21, 42, 55, 58, 59, 62, 64–67, 71–73]. Involvement in implementation most commonly included IHWs [18, 55, 58, 62, 63, 67, 70–73] and community members [20, 21, 42]. The Multimedia Messaging Service (MMS) clinic attendance trial, Blow Breathe Cough (BBC) program, and ECHS reportedly included First Nations people in program design [59, 61, 68] and the BBC program included First Nations people in program evaluation [61].

### Measures of program success

#### Outcome and output measures reported

Of the 27 identified articles, 23 stated indicators used to identify program success. Half of these articles stated outputs which included number of patients receiving various ear and hearing care services. Number of patients receiving services included those who received screening services [62, 70, 72, 74], ENT services [18, 60, 75], Child Nurse Specialist services [58], speech and language services [75] and unspecified audiology and follow-up services [18, 58, 69]. Other commonly utilised indicators included cost effectiveness [63, 64, 67], number of children identified as having an ear or hearing issue [58, 60, 62, 70, 74], and referral rates [62, 72–74]. One study utilised outputs regarding clinic attendance differences between groups in a randomised-control trial [68]. Seven studies included qualitative measures such as perspectives of perceived program impact [18, 55, 61, 65, 66, 75, 76]. One article reported outcome measures regarding surgical and hearing outcomes at post-surgical review [64]. Supplementary Table 2 captures study outputs and outcomes.

#### Funding and other sustainability factors reported

Most of the studies identified that programs relied on government funding mechanisms which could either ensure sustainability or lead to program discontinuation [18, 54, 55, 57–60, 63, 65]. For example, while the Healthy Ears, Better Hearing, Better Listening (HEBHBL) program depended on Australian Government funding, annual funding timing created logistical problems in the continuity of the service [18, 55]. The HoHEBP was funded through philanthropic organisations, community donations, and community fund-raising events which raised seed funding [69]. The AFHCAN program was funded with a $30 million grant in 1998 through the Alaska Federal Health Care Partnership [42]. The HOP received sustainable funding from the Ministry of Health and Social Services to guarantee continuity of the services [20, 21]. An ENT program found that use of telehealth for post-operative review was cost and time efficient, however identified the need for ongoing funding to expand the program [64]. The HEALS program was not able to be continued as an ongoing and sustainable service due to tight funding deadlines and lack of recurrent funding [75, 76]. The LiTTLe Program received funding from the Honda Foundation, Ian Thorpe Fountain for Youth, and the Federal Government’s Communities for Children Program, however, the program was discontinued due to a government funding reduction [65].

The mobile screening and surveillance service identified that sustainability of these programs was due to their cost-effectiveness, close alignment and integration with existing community services, and ongoing community consultation participation [62, 67, 71–73]. The Dangerous Decibels Program also found that community participation contributed to program self-sustainability [66]. The ear health screening program in NSW Australia was found to be more sustainable when Aboriginal project officers were trained to take on additional duties [70].

## Discussion

This scoping review aimed to identify key strategies, areas of focus relative to the care pathway, and factors that could ensure or threaten a program’s sustainability for First Nations children in high-income colonial-settler countries. Programs identified indicated three main strategies; (i) connecting patients to specialist services [18, 20, 21, 42, 55, 56, 58–60, 62–64, 67, 70–74], (ii) ensuring cultural safety of services [18, 20, 21, 42, 55, 56, 58, 59, 62, 63, 67–69, 71–73, 75, 76], and (iii) increasing entry into ear and hearing care pathways through screening or education/awareness programs [18, 54, 56, 58, 61, 65, 66, 69]. Connecting patients to care was achieved through outreach or mobile health clinics [18, 56, 58, 59, 62, 67, 69, 71–73], telehealth services or arranging and funding patient transportation [42, 58, 60, 62–64, 67, 70–73]. Ensuring cultural safety of services was achieved by employing and/or upskilling local community IHWs [18, 20, 21, 55, 56, 58, 59, 62, 63, 67, 69, 71–73] or providing cultural awareness training programs to non-IHWs [42]. Whereas increasing entry into ear and hearing care pathways was achieved by increasing access to care pathways with targeted or community-based screening programs [18, 20, 21, 42, 54, 55, 57–60, 62, 67, 69–74] or education and awareness programs [18, 54, 56, 58, 61, 65, 66, 69].

The current scoping review found that focal points of the identified programs were concentrated on early detection of ear disease and hearing loss as well as management of ear conditions through specialist services. Programs focused on achieving detection through raising awareness of ear disease and hearing loss symptoms, and conducting surveillance and screening [18, 20, 21, 42, 54–62, 65–67, 69–74]. The main goal of these programs was to provide a first point of access to the care pathway (i.e., bringing Aboriginal children into the pathway). Programs also focused on management through specialist services, namely ENT services, which frequently sought to connect patients to care by telehealth or arranging and paying for patient travel [18, 63, 64]. It is important that the concentration of these programs suggest that these are important and high areas of need. Yet discrete programs can increase fragmentation of pathways and reduce opportunities for children and families to navigate across different services and sectors (i.e. health, social and education systems). Systems thinking and systems modelling to improve complex healthcare services are emerging as a methodological approach to identify and understand interdependencies across and within services and sectors. This type of approach is important in embedding targeted programs like those described here to be effectively embedded within care pathway [78, 79].

Many programs aimed to ensure cultural safety of services through involvement of IHWs [18, 55, 58, 62, 63, 67, 70–73] or community members [20, 21, 42] in service delivery. While these factors are important, they appear limited when considered through the lens of a contemporary understanding of cultural safety. First Nations people have the right to govern their own health matters across system levels, yet current approaches limit partnership with First Nations people and focus only on program implementation [80]. Future programs should ensure First Nations people are partnered with in program design where cultural safety is considered throughout all stages, the system is designed for the people who it serves [81], and the likelihood of program sustainability is increased.

Programs which included IHWs in service delivery may, in addition to achieving cultural safety, have been aiming to bolster sustainability. Although further research is needed, inclusive dialogue with IHWs has been recognised as contributing to sustainable workplace environments [82]. Although many programs reported First Nations involvement, this factor was reported in relation to sustainability of programs by only three studies [66, 70, 71]. Long-term sustainability requires dedicated funding revenues, and it was clear that not all programs were able to show this. In fact, one program was discontinued due to reduction in funding [65]. Other programs may be at risk of discontinuation, relying primarily on philanthropic funding and community fund-raising [69]. Therefore, embedding programs into existing policies and funding streams is important to ensure their viability in the long-term.

The current scoping review indicates that the majority of programs focus on output measures, presumably a by-product of a discrete program (capturing an episode of care) rather than a program embedded within a connected system of care. Output measures give an indication of program reach but assess only health system qualities, while outcome measures evaluate to long-term goals. Outcome measures regarding ear and hearing care should include mitigation of ear disease and hearing loss impacts with respect to wellbeing [37], speech and language skills [83], academic attainment [31], and employment [41]. Further outcome measures may include family understanding or confidence in supporting their child and satisfaction with cultural safety of service delivery. It is acknowledged that measuring comprehensive outcomes is a laudable yet challenging goal. These often require within- and cross-sector partnerships however, they provide a robust measure of the impact/s of prevention programs.

Due to inconsistent study reporting, the current scoping review was unable to fully capture population-based demographic data of participants who received care. Rather, program location and setting were consistently reported and captured in this review. The high number of programs (*n* = 12) which addressed geographical access as a key barrier to care, indicates that many programs focused on servicing First Nations people residing in rural, regional or remote areas. This is presumably due to issues such as inequitable workforce distribution and extended wait times [84] that frequently hinder access to care for First Nations populations in these areas. It is worth noting however that the majority of First Nations people reside in urban or metropolitan areas [85–90] and these populations are not immune to socio-economic disadvantage or pervasive health inequity [75].

### Study limitations

Findings should be interpreted with caution because whilst the scoping review utilised database search strategies to capture eligible articles on programs in Australia, New Zealand, Canada and the United States, most studies found were Australian programs. Only one program in Canada and two in Alaska were identified. No programs were identified as operating in New Zealand. Given the search terms utilised in the search strategy were inclusive of First Nations terms in New Zealand, we conclude that First Nations specific programs in these countries are scarce and/or unreported upon in both formal and grey literature. The methodological approach taken required program information to be publicised, hence information provided verbally through stakeholders could not be used to populate the results tables. This review aimed to capture reported measures of program success and although this information was not always reported in publications does not necessarily mean no data was captured.

## Conclusion

Substantial efforts have been made to provide ear and hearing care to First Nations children to address the high burden of otitis media and hearing loss. However, discrete programs can limit long-term viability given the need to seek or advocate for funding on an ongoing basis. While they might solve one part of the pathway, failing to address other significant gaps could potentially limit their longer-term benefit to the child and family. As the Lancet Global Health Commission on High Quality Health Systems in the SDG Era states, “high quality health systems should be informed by four values: they are for people, and they are equitable, resilient and efficient” [81]. Therefore, future programs should work with First Nations community across all stages of the program development, implementation, and evaluation stages to ensure that they are tailored for the people they are designed for. They should be evaluated with outcome measures that demonstrate they are equitable and efficient at mitigating ear disease, hearing loss and their longer-term effects. Importantly, they should be resilient whereby factors which affect the sustainability of programs should be a primary focus of the planning, design and implementation.

## Supplementary Information

**Additional file 1: Supplementary Table 1.** Program activities. *Aboriginal refers to Aboriginal and Torres Strait Islander peoples.

**Additional file 2: Supplementary Table 2.** Program core elements. *Aboriginal refers to Aboriginal and Torres Strait Islander peoples.

## Data Availability

All data generated or analysed during this study are included in this published article.

## Abbreviations

AHW: Aboriginal Health Worker
AFHCAN: Alaska Federal Health Care Access Network
BBC: Blow Breathe Cough
ENT: Ear, Nose, and Throat
ECHS: Enhanced Child Health Schedule
HoHEBP: Hear Our Heart Ear Bus Project
HOP: Hearing and Otitis Program
HEALS: Hearing EAr health Language and Speech services
HEBHBL: Healthy Ears, Better Hearing, Better Listening
IHW: Indigenous Health Worker
MMS: Multimedia Messaging Service
NSW: New South Wales
NT: Northern Territory
QLD: Queensland

## Footnotes

1 First Nations people is the term the authors chose as a collective term to describe the original inhabitants of Australia, New Zealand, Canada, and the United States. The authors acknowledge that opinion and preference on this matter may vary between country, community, and individual.
