# Supplementary material for: Ear and hearing care programs for First Nations children: a scoping review
Source: BMC Health Serv Res. 2023 Apr 19;23:380. doi: 10.1186/s12913-023-09338-2 (PMC10116763; doi:10.1186/s12913-023-09338-2)
Supplement: Supplementary file 1 — Additional file 1: Supplementary Table 1. Program activities. *Aboriginal refers to Aboriginal and Torres Strait Islander peoples. [file 12913_2023_9338_MOESM1_ESM.docx]

| **Supplementary Table 1 Program activities** | | | | | | |
| --- | --- | --- | --- | --- | --- | --- |
| **Program/activity name** | **First author, date** | **Years active** | **State, country** | **Setting** | **Population** | **Program/activity details** |
| Hearing Health Outreach Program | AIHW, 2019 | 2007 - 2018 | NT, AUS | Community. | Aboriginal children and young people. | Health education, promotion and prevention activities provided by the public health sector. |
| Deadly Ears | AIHW, 2021 | 2007 – 2021 | QLD, AUS | Community. | Aboriginal children and young people. | Ear and hearing assessments provided for Aboriginal children (≤4 years prioritised). |
| Healthy Ears, Better Hearing, Better Listening | Siggins Miller, 2017  Department of Health and Ageing 2013 | Current | QLD, NT, ACT, SA, WA, NSW, AUS | Pre-schools, schools. | Aboriginal children and young people. | Outreach health services and activities prioritising locations of highest need. |
| Surgical support | Siggins Miller, 2017 | Current | QLD, SA, VIC, WA, AUS | Community. | Aboriginal clients (all ages). | Outreach ENT surgery delivered to rural and remote areas (funding travel costs for patient and clinician). |
| Care for Kids Ears | Siggins Miller, 2017  Australian Government Department of Health and Ageing, 2013 | Current | AUS (national) | Not applicable. | Parents, carers, early childhood educators, teachers and health professionals. | Online resources for ear health awareness distributed to parents and carers of Aboriginal children. Teachers, early childhood and community groups, and health professionals receive resource kits. |
| Hearing Otitis Program | Billard, 2014  Ayukawa, 2014 | 1985 - current | Nunavik, CA | Community. | Residents of Nunavik (95% Inuit ancestry). | Culturally appropriate audiology services provided by Inuit specific roles such as the siutilirijiit (“the ones who know about ears”) and the aaniasiurtiapiit (“little nurse”). |
| Blow, Breathe, Cough Program | Doyle, 2010 |  | WA, AUS | School. | Children aged 4 – 8 years old (Majority Aboriginal). | Program activities incorporate deep breathing, coughing, physical movement and blowing the nose. |
| Mobile telemedicine-enabled screening service | Nguyen, 2015  Elliott, 2012  Smith, 2012  Smith, 2013  Smith 2015 | 2009 - 2014 | QLD, AUS | School. | Aboriginal children (no age range given). | AHWs used asynchronous telehealth in a mobile clinic for remote ENT evaluation of otoscopy, tympanometry and audiometry. |
| Enhanced Child Health Schedule | Government of WA Country Health Service, 2020 | June 2018-19 | WA, AUS | Not stated. | Aboriginal children (no age range given). | Ear and hearing health checks, including otoscopy, audiometry, and tympanometry. |
| Schedule of hearing and ear health screening | Government of WA Child and Adolescent Health Service, 2014 | Current | WA, AUS | School settings, General Practice. | Children aged 0 – 5 years (including Aboriginal children). | All children receive screening questions at intervals of 0-14 days, 8 weeks, 4 months, 12 months, and 2 years. From 8 weeks, Aboriginal children receive otoscopy and tympanometry (in addition to screening questions). At school entry, all children receive screening questions, otoscopy, and audiometry, while additionally Aboriginal children receive tympanometry. Annual ear health screening for Aboriginal children in Pre-primary and Year 1 and opportunistic screening encouraged for Aboriginal children. |
| Hear our Heart Ear Bus Project | Rees, 2014 | Current | NSW, AUS | Childcare centres, primary and secondary schools, community health centres, preschools, AMSs. | Aboriginal children (no age range given). | Targeted hearing screening (otoscopy, tympanometry, audiometry) in mobile clinic (and community day clinics to capture children not attending school) for children “at risk” identified by teachers. Based on results, diagnostic tests provided. Health promotion provided to teachers and community. |
| Alaska Federal Health Care Access Network program | Robler 2018 | 2001 - Current | AK, US | Community. | All Alaskan residents but high population of Alaskan Natives. | Healthcare including audiology, cardiology, dental, dermatology, and otolaryngology. Local community providers/health aides facilitate in-person consultations and audiologists remotely assess video otoscope, audiometer and tympanometer. Real time telehealth consultation frequently utilised. |
| ENT Program | Jacups, 2017 | 2012 - 2016 | NT, AUS | Private hospitals. | Aboriginal children aged 0-15 years. | Children expedited from waitlists for ENT surgery with travel expenses covered. Follow up services facilitated via telehealth for virtual consultation and ear assessment. Post-operative audiology performed in patient’s home by an Apunipima audiologist. |
| ENT model | Jacups, 2018 | Current | NT, AUS | Public hospitals. | Aboriginal children (no age range given). | Patients travel to a regional centre for ENT surgery at a public hospital with support from an ENT coordinator. |
| LiTTLe Program | Jones, 2018 | 2006 - 2014 | NT, AUS | Community. | Aboriginal children aged 0 - 3 years. | Parents encouraged and supported to speak to their child to promote language learning and school readiness. |
| Dangerous Decibels Program | Martin, 2017 | 2010–Current | AK, US | Community. | 4^th^ and 5^th^ grade students. | Local media, classroom education, family and community outreach, and web-based activities implemented to promote noise-induced hearing loss and tinnitus prevention. |
| Multi-media messages clinic attendance trial | Phillips, 2014 | 2010 | NT, AUS | Community. | Aboriginal children aged 13 years or under (separated into intervention and control arms). | Multi-media health messages in local languages were sent to an intervention group over 6 weeks. |
| Ear health screening program | Reeve, 2014 | 2014 | NSW, AUS | School. | School children in the Fitzroy Valley (high Aboriginal population). | Otoscopy, audiometry and tympanometry performed and if necessary, referrals for ENTs provided. Telehealth-enabled ENT reviews conducted. |
| Australian Nursing Student-led School Vision and Hearing Screening Program | Tolchard, 2017 | Undisclosed | Regional Australia (state undisclosed) | School. | Elementary school children in a high Aboriginal population. | Clinical student nurses conducted six ear and eye screening events over 2-month period. Referrals and prescriptions provided by nurse practitioner. |
| Hearing EAr health Language and Speech services project | Young, 2016  Young, 2017 | 2013 – 2014 | NSW, AUS | Aboriginal Community Controlled Health Services. | Aboriginal children aged 0-17 years. | ENT surgery and speech pathology services. |
| NSW Ear Health Program | NSW Health, 2011 | Current | NSW, AUS | Not stated. | Aboriginal children, their parents, carers and wider community. | Ear health surveillance implemented within existing programs. |
