# Supplementary material for: Ear and hearing care programs for First Nations children: a scoping review
Source: BMC Health Serv Res. 2023 Apr 19;23:380. doi: 10.1186/s12913-023-09338-2 (PMC10116763; doi:10.1186/s12913-023-09338-2)
Supplement: Supplementary file 2 — Additional file 2: Supplementary Table 2. Program core elements. *Aboriginal refers to Aboriginal and Torres Strait Islander peoples. [file 12913_2023_9338_MOESM2_ESM.docx]

| **Supplementary Table 2 Program core elements** | | | | |
| --- | --- | --- | --- | --- |
|  | **Program strategies** | | **Measures of sustainability and success** | |
| **Program/activity name** | **Specific strategies employed** | **Extent of First Nations involvement** | **Funding and other sustainability factors reported** | **Outcome and output measures reported** |
| Hearing Health Outreach Program | Telehealth; Clinical Nurse Specialists to increase coordination and service linkage; Upskilling local AHWs. | *Implementation:* AHWs provided audiology services; Aboriginal community hearing workers trained in hearing health and prevention. | Federal government funded. | *Outputs:*  No. of audiology services provided, children who received Child Nurse Specialist services, and children with hearing loss or condition.  No. of preventative hearing health promotion or training services/activities. |
| Deadly Ears | Telehealth. | Not stated. | State government funded. | *Outputs:*  No. of audiology services provided, patients who received ENT clinic services or surgery, and patients with hearing loss.  Proportion of patients with at least 1 ear condition; Most common type of identified ear condition. |
| Healthy Ears, Better Hearing, Better Listening | Not stated. | *Implementation:* Health services provided by health professionals including AHWs. | Federal government funded.  Annual funding timing creates logistical problems. | *Outputs:*  No. of patients who received services. |
| Surgical Support | Patient and health professional financial support for travel and accommodation. | Not stated. | Federal government funded. | *Outputs:*  No. of children who received surgery.  *Qualitative measures:*  Patient satisfaction (data inconsistently captured); Health professionals, service providers, and stakeholder’s perspectives on program operation. |
| Care for Kids Ears | Resources available online (free of charge). | *Implementation:* AHWs among those utilising resource kits. | Federal government funded. | *Qualitative measures:*  Attitudes towards resource kits; Perspectives on enhancing online resources; Perceptions of ear disease within communities. |
| Hearing and Otitis Program | Strategic program location. | *Implementation:* Local community members trained for program roles. | Sustainable funding from the Ministry of Health and Social Services was given to guarantee continuity of the services. | Not stated. |
| Blow, Breathe, Cough Program | Prevention through hygiene education for school children. | *Design:* Questionnaire piloted with teachers, one AHW and child health nurses.  *Evaluation:* AHWs completed survey. | Not stated. | *Outputs:*  Response rate to questionnaires.  *Qualitative measures:*  Perspectives regarding benefits and barriers of programs, and what data (if any) was collected on program outcomes. |
| Mobile screening and surveillance service | Telehealth; Mobile clinic. | *Implementation:* AHWs conducted screening. | Cost effective; Close alignment and integration with existing community services; Ongoing community consultation participation. | *Outputs:*  Quality adjusted life year; Screening rates; Screening results; Referral rates; ENT feasibility ratings; Waiting times; No. of outpatient appointments and surgical procedures. |
| Enhanced Child Health Schedule | Additional home visiting contacts for high priority families. | *Design:* Aboriginal health staff included in consultation group.  *Implementation:* Ear checks often delivered by Aboriginal health nurses. | State government funded. | Not stated. |
| Schedule of hearing and ear health screening for WA children | Not stated. | Not stated. | State government funded. | Not stated. |
| Hear our Heart Ear Bus Project | Mobile clinic;  School staff receive professional development to assist identification of at-risk children; Community education and utilisation of Blow Breathe Cough Program resources. | Not stated. | Philanthropic organisations, community donations, and community fund-raising events. | *Outputs:*  No. of towns visited, patients who received services, and hearing tests conducted.  Referral rates; Percentage of Aboriginal to non-Aboriginal children requiring follow-up services, surgery, and hearing aids. |
| Alaska Federal Health Care Access Network program | Telehealth; Culturally competent audiologists; Program integrated into clinical practice at 248 locations. | *Implementation:* Local community providers and health aides facilitate in-person consultations. | Alaska Federal Health Care Partnership funded. | No evaluation or method/results provided, however general statistics on program commented on.  Telehealth reduced wait times by 59% for in-person otolaryngology consultation; Reduced need for patient travel for otolaryngological  care by 85%; Saved healthcare system approximately $500,000 in airfare. |
| ENT Program | Telehealth. | Not stated. | Telehealth for post-operative review was cost and time efficient, however ongoing funding would be required to expand the program. | *Outcomes:*  Surgical and hearing outcomes.  *Outputs:*  Cost efficiency; Wait times. |
| ENT model | Telehealth. | *Implementation:* Health management may be facilitated by AHWs. | State government funded. | *Outputs:*  Incremental cost difference between three service delivery models. |
| LiTTLe Program | Not stated. | *Implementation:* Aboriginal community members provided support as program workers. | Program discontinued due to reduction in  government funding.  Funded by: Honda Foundation, Ian  Thorpe Fountain for Youth, and federal government’s  Communities for Children Program. | *Qualitative measures:*  Perspectives on program implementation as well as speech and language strategies, hearing health and intervention, school readiness, and ideas for the future. |
| Dangerous Decibels Program | Community supported promotion of hearing health education. | *Implementation:* Community member participation from American Indian communities. | Community participation contributed to program self-sustainability. | *Qualitative measures:*  Community awareness and changed habits post-implementation. |
| Multimedia Messaging Service clinic attendance trial | Multi-media health messages in local languages encourage clinic attendance. | *Design:* The design of messages determined in consultation with local Aboriginal teachers and interpreters. | Not stated. | *Outputs:*  Clinic attendance; Identified ear conditions. |
| Ear health screening program | Telehealth. | *Implementation:* Follow-up services provided by AHWs. | To maintain sustainability, Aboriginal project officers upskilled to take on referral and telehealth session administration duties. | *Outputs:*  Screening rates; ENT review times; Primary care management (with reference to guidelines). |
| Australian Nursing Student-led School Vision and Hearing Screening Program | Not stated. | Not stated. | Not stated. | *Outputs:*  Screening rates; Referral rates; Eye and ear presentations. |
| Hearing EAr health Language and Speech services project | Free services in metropolitan area. | *Implementation:* Aboriginal Community Controlled Health Organization partnership. | Tight funding deadlines; Lack of recurrent funding. | *Outputs:*  No. of children who received speech and language pathology sessions, and ENT procedures.  *Qualitative measures:*  Healthcare provider and parent/caregiver  perspectives on perceived program impact. |
| NSW ear health program | Prevention using broad public health approach. | Not stated. | State government funded. | Not stated. |
